# Screening performance of sternal CT attenuation for opportunistic osteoporosis

**DOI:** 10.1186/s12891-026-10181-9

**Published:** 2026-07-09

**Authors:** Rongkang Liu, Chonggang Chen, Wenhua Wu, Lijiang He

**Affiliations:** Department of Spinal Surgery, The Second Affiliated Hospital of Fujian Medic al University, No. 34, Zhongshan North Road, Licheng District, Quanzhou City, 362000 Fujian Province China

**Keywords:** Osteoporosis, Opportunistic screening, Computed Tomography (CT), Sternal CT HU value, Bone mineral density (BMD)

## Abstract

**Background:**

Osteoporosis represents a major global public health problem, yet it remains substantially underdiagnosed. Although dual-energy X-ray absorptiometry (DXA) is considered the clinical gold standard for bone mineral density (BMD) assessment, its routine use for population-based screening is limited. As an alternative, the use of routine computed tomography (CT) for opportunistic screening has emerged as a promising approach. This study aims to evaluate the feasibility of utilizing sternal CT values (Hounsfield units, HU) from chest CT scans to assess osteoporosis.

**Methods:**

This retrospective study included 145 patients aged ≥ 50 years who underwent chest CT and DXA within a one-month interval. CT HU values were measured at the upper, middle, and lower levels of the sternum by three independent observers. Inter-observer reliability was assessed using intraclass correlation coefficients (ICC). Correlations between sternal CT values, T-score, age, and body mass index (BMI) were analyzed using Pearson correlation. Receiver operating characteristic (ROC) curve analysis was performed to evaluate the diagnostic performance of sternal CT HU values for identifying osteoporosis.

**Results:**

Significant positive correlations were observed between sternal CT HU values and DXA T-score at all measured levels (*r* > 0.506, *P* < 0.01), with the mean sternal CT value exhibiting the strongest association (*r* = 0.632). Inter-observer agreement was excellent across all sternal levels (ICC range: 0.926–0.980). Moreover, ROC analysis revealed that the lower sternal level provided the best diagnostic performance for osteoporosis, with an area under the curve (AUC) of 0.8712 (95% CI: 0.8128–0.9296). An optimal cutoff value of 64.90 HU was identified, with a sensitivity of 80.9% and a specificity of 85.7%.

**Conclusions:**

Sternal CT HU values measured on chest CT scans are significantly correlated with DXA-measured BMD. In particular, measurements at the lower sternal level showed the best diagnostic performance and may serve as a potential supplementary tool for opportunistic osteoporosis screening in elderly patients.

## Introduction

Osteoporosis is a common age-related skeletal disorder and represents a major global public health concern [1]. It is estimated that more than 200 million people worldwide are affected by osteoporosis, with the incidence expected to rise further due to global population aging. The disease is characterized by reduced bone mass and deterioration of bone microarchitecture, leading to increased bone fragility and a heightened risk of fractures [2]. Osteoporotic fractures represent the most severe clinical outcome of osteoporosis and are associated with increased morbidity, functional impairment, and reduced quality of life, thereby imposing a considerable burden on healthcare systems worldwide [3]. However, osteoporosis is frequently underrecognized in clinical practice, as many individuals remain undiagnosed until a fragility fracture occurs [4].

Currently, DXA is regarded as the gold standard for the clinical diagnosis of osteoporosis and the assessment of fracture risk [5]. However, its widespread implementation is subject to several important limitations. The availability of DXA equipment remains limited outside of tertiary care centers, which restricts its use as a widespread screening tool [6]. Additionally, DXA screening is not routinely performed unless specifically indicated, which may result in missed opportunities for identifying individuals at elevated fracture risk. Besides, owing to its two-dimensional projection-based nature, DXA cannot distinguish between trabecular and cortical BMD and is susceptible to interference from degenerative spinal changes [7]. Quantitative computed tomography (QCT) has been shown to be superior to DXA for the assessment of spinal osteoporosis, as it offers volumetric measurements that are largely independent of degenerative lesions, such as osteophytes, as well as vertebral cortical bone [8, 9]. While QCT offers high accuracy, its utility as a population-level screening tool is precluded by its relatively high radiation dose and cost. Consequently, there is an urgent clinical need for an effective, practical, and cost-efficient approach to identify individuals at high risk for osteoporosis.

In recent years, the concept of opportunistic screening has gained increasing attention in the assessment of osteoporosis [10]. Many patients undergo CT examinations for indications unrelated to bone health, such as pulmonary nodule surveillance, cardiovascular evaluation, or trauma assessment. BMD can be evaluated on these routine CT scans by measuring CT HU values at specific anatomical sites [11–14]. Among these, chest CT is one of the most commonly performed scans in clinical practice and therefore provides a large and readily accessible imaging data source for opportunistic osteoporosis screening.

Most previous studies have evaluated BMD by measuring CT HU values in the thoracic or lumbar spine [15–18]. However, these regions are frequently affected by degenerative changes, osteophyte formation, and vertebral compression fractures, which may compromise the accuracy of bone assessment using CT. Notably, the sternum is a flat bone that is routinely included in the field of view of chest CT examinations and is less affected by the degenerative changes described above. Therefore, it may serve as a promising alternative site for opportunistic osteoporosis screening.

Previous work [19] has shown that sternal CT attenuation value correlates with BMD. However, methods relying on advanced segmentation are not suited for routine practice, and the diagnostic value of different sternal levels remains unclear. Therefore, this study aims to identify the most informative sternal level and evaluate the diagnostic performance of simple CT measurements at the upper, middle, and lower sternum on routine chest CT scans, using DXA as the reference standard.

## Methods

### Study participants

The requirement for written informed consent was waived because of the retrospective design of this study. This study included patients who visited the Department of Orthopedics at the Second Affiliated Hospital of Fujian Medical University between January 1, 2023, and June 1, 2024. The inclusion criteria were as follows: age ≥ 50 years; availability of both chest CT and DXA examinations performed within a one-month interval; and complete clinical data. Exclusion criteria were as follows: (1) sternal infectious lesions, primary or metastatic malignancies, prior sternal surgery, or confirmed sternal fractures; (2) severe metabolic bone diseases, including advanced renal insufficiency or hyperparathyroidism [20]; (3) recent or long-term use of glucocorticoids or other medications known to substantially affect bone metabolism; (4) inadequate chest CT image quality due to pronounced motion or metallic artifacts; and (5) severe spinal degenerative changes or a history of spinal surgery. Ultimately, 145 patients were enrolled. Baseline demographic data, including age, sex, height, weight, and BMI, were collected for all participants.

### DXA scanning

The BMD was measured in all study participants using DXA (Discovery-A multi‑detector X-ray bone densitometer; Hologic, Marlborough, Massachusetts, USA). The measurement sites included the lumbar spine (L1–L4) and the hip (total hip and femoral neck). According to the World Health Organization (WHO) diagnostic criteria, patients were categorized according to their T-score: normal (T-score ≥ -1.0), osteopenia (-2.5 < T-score < -1.0), and osteoporosis (T-score ≤ -2.5) [21]. The final diagnosis was determined by the lowest T-score observed among the lumbar spine, total hip, and femoral neck.

### CT scanning

Thoracic CT images were acquired using a multi-detector CT scanner (Brilliance 64, Philips, Amsterdam, The Netherlands), with patients positioned supine in a head-first orientation. The scan range extended from the thoracic inlet to the costophrenic angles. The acquisition parameters were as follows: tube voltage, 120 kV; automatic tube current modulation (maximum 240 mA); matrix, 512 × 512; pitch, 0.8; rotation time, 0.5 s/r; slice thickness, 1.0 mm; and reconstruction interval, 1.0 mm. All scans were performed without intravenous contrast administration. All images were archived in the Picture Archiving and Communication System (PACS) for subsequent analysis.

**Fig. 1:**
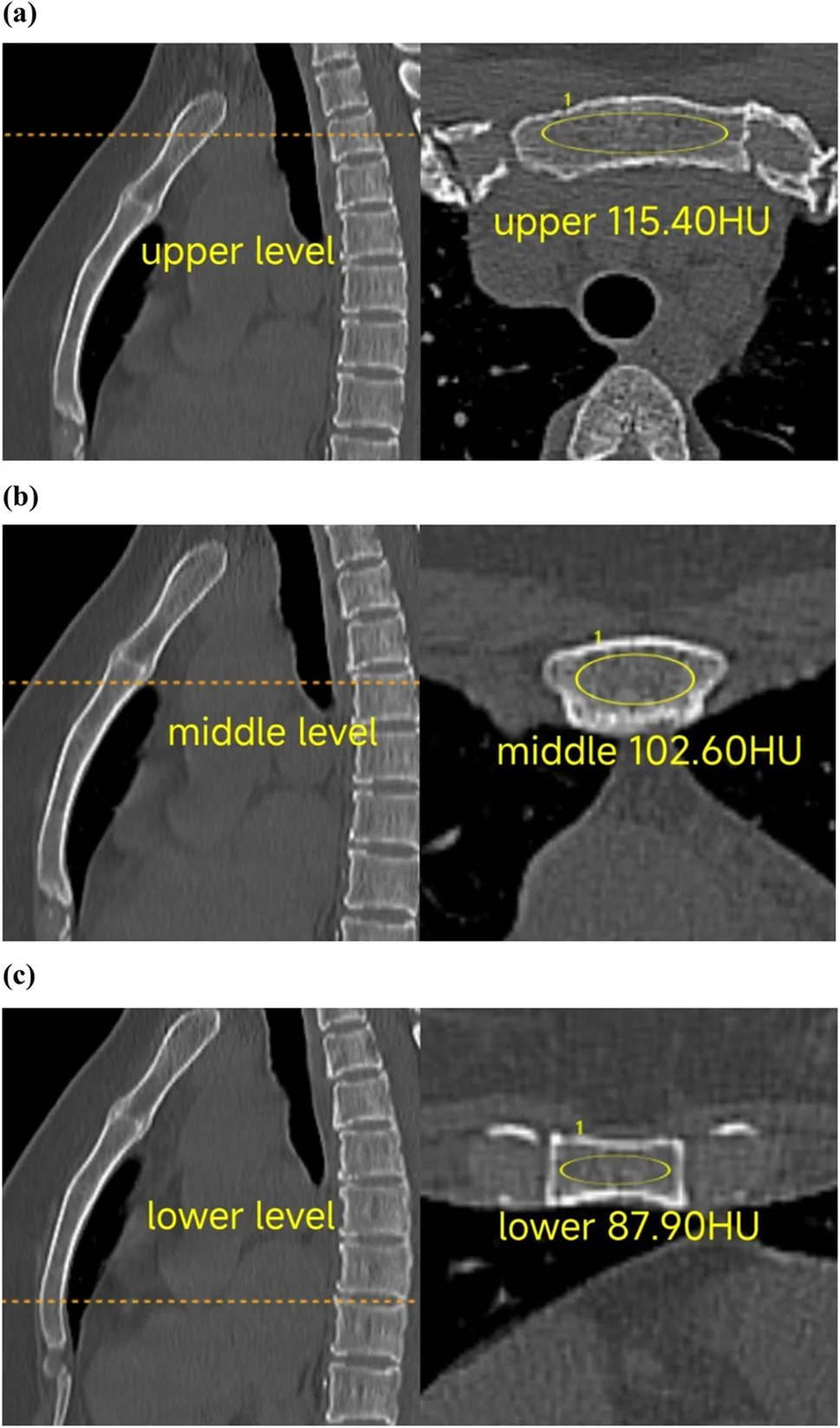
Methods for measuring CT values at different levels on chest CT scans. **a** CT scan of upper level of the sternum in the sagittal plane. **b** CT scan of middle level of the sternum in the sagittal plane. **c** CT scan of lower level of the sternum in the sagittal plane

Three independent orthopedic surgeons, blinded to the DXA results, measured the CT HU of the sternum. All three observers completed standardized training on sternal anatomy, ROI placement, cortical bone avoidance, and bone window settings prior to measurement. Duman et al. [22] measured the CT HU by placing a region of interest (ROI) within the trabecular bone of the sternal body on axial sections. Given the sternum’s anatomical complexity, comprising the manubrium, body, and xiphoid process with varying thicknesses, we hypothesized that the CT HU values and their diagnostic efficacy for osteoporosis might differ across anatomical levels [23]. Therefore, CT HU values were measured at the upper, middle, and lower levels of the sternum. The upper level was defined just below the sternoclavicular joint, and measurements were obtained from 2 to 3 consecutive slices inferior to the joint. The middle level was located at the junction of the manubrium and sternal body, with measurements taken from 2 to 3 consecutive slices inferior to this junction. The lower level was defined just above the xiphoid process, and measurements were obtained from 2 to 3 consecutive slices superior to this level. At each level, an elliptical ROI was placed within the central cancellous bone, carefully avoiding the cortical margins (Fig. 1). To minimize partial volume effects, the middle slice was centered at the level with the largest cancellous area. The ROI size was adapted to the individual anatomy and the specific sternal level, typically ranging from approximately 50 to 150 mm², aiming to cover as much cancellous bone as possible. All ROI placements were performed using bone window settings (window width: 1500 HU; window level: 450 HU) to ensure clear visualization of cortical boundaries. At each level, the HU value for that level per observer was calculated as the average of three consecutive slices.

For each patient, all three observers independently performed ROI placement at each sternal level. The three measurements at each level were then averaged to obtain a single HU value for the upper, middle, and lower sternum per patient. These averaged values were used for all subsequent analyses. The “Sternal-Average” was subsequently calculated as the mean of these three level-specific values. Although measurements were performed by orthopedic surgeons, approximately 30% of the cases (*n* = 44) were randomly sampled and reviewed by a senior musculoskeletal radiologist, who was blinded to the DXA results, to ensure data quality.

### Statistical methods

Statistical analysis and data visualization were performed using SPSS version 26.0 and GraphPad Prism version 10.1.2. Continuous variables are presented as mean ± standard deviation (SD) for normally distributed data, or as median and interquartile range (IQR) for non-normally distributed data. Categorical variables are expressed as frequencies and percentages. Differences in age, BMI, T-score (minimum of L1-L4, total hip, and femoral neck T-scores), and CT values across the three groups were compared using one-way ANOVA; post-hoc pairwise comparisons were conducted using the LSD-t test for homogeneous variances or the Games-Howell test for non-homogeneous variances. Categorical data were analyzed using the Chi-square test. Inter-observer reliability among the three surgeons was assessed using the intraclass correlation coefficient (ICC), with an ICC > 0.90 indicating excellent consistency. Pearson correlation analysis was used to assess the relationships between sternal CT values and age, BMI, and T-score. To determine the diagnostic performance of sternal CT values in identifying osteoporosis (using DXA as the gold standard), ROC curves were generated, and the area under the curve (AUC) was calculated. The optimal cutoff values were determined using the Youden index, and corresponding sensitivity and specificity were calculated. Statistical significance was set at *P* < 0.05. To evaluate the independent diagnostic contribution of sternal CT HU values for osteoporosis, univariate and multivariate logistic regression analyses were performed. The dependent variable was the presence of osteoporosis (coded as 1 for osteoporosis, 0 for no osteoporosis). Sternal CT HU values at each level (upper, middle, and lower) were separately entered into the models, with age, BMI, and gender included as covariates. The results are presented as odds ratios (OR) with 95% confidence intervals (CI).

## Results

### Clinical baseline data

Based on the DXA results, 145 patients were categorized into three groups: normal bone mass (*n* = 10, 6.9%), osteopenia (*n* = 46, 31.7%), and osteoporosis (*n* = 89, 61.4%). The cohort consisted of 110 women (75.9%) and 35 men (24.1%), with no significant difference in sex distribution across the three groups (*P* = 0.087). Statistical analysis revealed no significant differences in sex distribution across the groups. However, age, BMI, and T-score of the lumbar spine and femoral neck differed significantly among the groups (*P* < 0.05).

Specifically, the mean age progressively increased from the normal group (61.8 ± 9.8 years) to the osteopenia group (66.0 ± 7.0 years) and the osteoporosis group (70.6 ± 8.9 years). In contrast, BMI and lumbar spine T-score showed a decreasing trend across these groups. The normal group had the highest femoral neck T-score (− 0.4 ± 0.4), whereas the lowest values were observed in the osteopenia group (− 2.4 ± 0.9). Detailed baseline characteristics are summarized in Table 1.

**Table 1:** Comparison of clinical baseline data, L1-L4 T-score and Neck T-score in the three groups

Features	normal(*n* = 10)	Osteopenia(*n* = 46)	Osteoporosis(*n* = 89)	*P*-value
Age	61.8 ± 9.8	66.0 ± 7.0	70.6 ± 8.9	<0.05
BMI	27.2 ± 2.9	26.2 ± 3.9	23.6 ± 4.0	<0.05
Women (%)	7(70.0%)	30(65.2%)	73(82.0%)	0.087
L1-L4 T‐score	0.6 ± 1.2	-0.8 ± 0.7	-2.8 ± 0.8	<0.05
Neck T-score	-0.4 ± 0.4	-1.4 ± 0.6	-2.4 ± 0.9	<0.05

### Measurement consistency and intergroup comparison of sternal CT values

Table 2 presents the measurement reliability and inter-group comparisons of sternal CT values. The ICC for the three observers across all sternal levels—upper, middle, lower, and average—were exceptionally high, ranging from 0.926 to 0.980, with all lower bounds of the 95% confidence interval (CI) > 0.90. These results confirm that our measurement protocol demonstrates excellent consistency and reproducibility.

**Table 2:** Comparison of sternal CT values at different levels and inter-observer agreement

	Total (*n* = 145)	Normal (*n* = 10)	Osteopenia(*n* = 46)	Osteoporosis(*n* = 89)	F-value	*P*-value	ICC (95% CI)
Sternal-Upper	91.3 ± 11.0	101.2 ± 12.5	97.2 ± 9.9	87.1 ± 9.2	21.928	<0.05	0.954(0.940-0.966)
Sternal-Middle	74.3 ± 8.6	82.3 ± 13.5	78.8 ± 7.3	71.0 ± 6.8	23.475	<0.05	0.926(0.903–0.945)
Sternal-Lower	64.4 ± 9.2	74.9 ± 9.4	70.4 ± 7.5	60.1 ± 7.1	40.422	<0.05	0.949(0.933–0.962)
Sternal-Average	76.7 ± 8.4	86.5 ± 11.5	82.1 ± 6.8	72.7 ± 6.0	40.348	<0.05	0.980(0.974–0.986)

F-value represents the test statistic from one-way ANOVA comparing sternal CT HU values across the three groups (normal, osteopenia, and osteoporosis)

One-way ANOVA revealed significant differences in sternal CT HU values across the three BMD categories at all anatomical levels (all *P* < 0.05). Specifically, taking the upper sternal level as an example, the normal bone mass group exhibited the highest CT HU values (101.2 ± 12.5 HU), followed by the osteopenia group (97.2 ± 9.9 HU), with the lowest values observed in the osteoporosis group (87.1 ± 9.2 HU). This declining trend in CT HU values across the three BMD categories (normal → osteopenia → osteoporosis) was consistent across all measured levels.

Post-hoc pairwise comparisons using the LSD method revealed that the normal group had significantly higher CT HU values at all levels compared to both the osteopenia and osteoporosis groups (all *P* < 0.05). However, no statistically significant differences were found between the osteopenia and osteoporosis groups at any sternal level or for the average CT HU value (all *P* > 0.05). 

### Correlations between age, BMI, T-score value, and Sternal HU

Table 3 shows the correlations between sternal CT HU values and BMD, age, and BMI. Pearson correlation analysis revealed significant positive correlations between sternal CT HU values and DXA-derived T-score at all measured levels: upper sternum (*r* = 0.546, *P* < 0.01), middle sternum (*r* = 0.506, *P* < 0.01), lower sternum (*r* = 0.605, *P* < 0.01), and average sternal HU (*r* = 0.632, *P* < 0.01). Specifically, the average sternal CT HU value exhibited the strongest correlation (*r* = 0.632, *P* < 0.01), suggesting that it may be the most reliable indicator of overall bone health.

**Table 3:** Correlations between age, BMI, T-score value, and Sternal HU at different levels

	Sternal-upper HU	Sternal-middle HU	Sternal-lower HU	Sternal-average HU
T-score	0.546^**^	0.506^**^	0.605^**^	0.632^**^
age	-0.268^**^	-0.323^**^	-0.307^**^	-0.340^**^
BMI	0.273^**^	0.201^*^	0.172^*^	0.251^**^

**: The correlation was significant at a 0.01 level (two-tailed)

*: The correlation was significant at a 0.05 level (two-tailed)

Additionally, a significant negative correlation was found between age and CT HU values across all sternal levels (r ranging from − 0.268 to -0.340, *P* < 0.01). This negative trend is consistent with the expected physiological decline in bone mass due to age-related osteoporosis [24, 25]. Furthermore, sternal CT HU values were positively correlated with BMI (r ranging from 0.172 to 0.273, *P* < 0.05).

### ROC analysis of diagnostic models

**Fig. 2:**
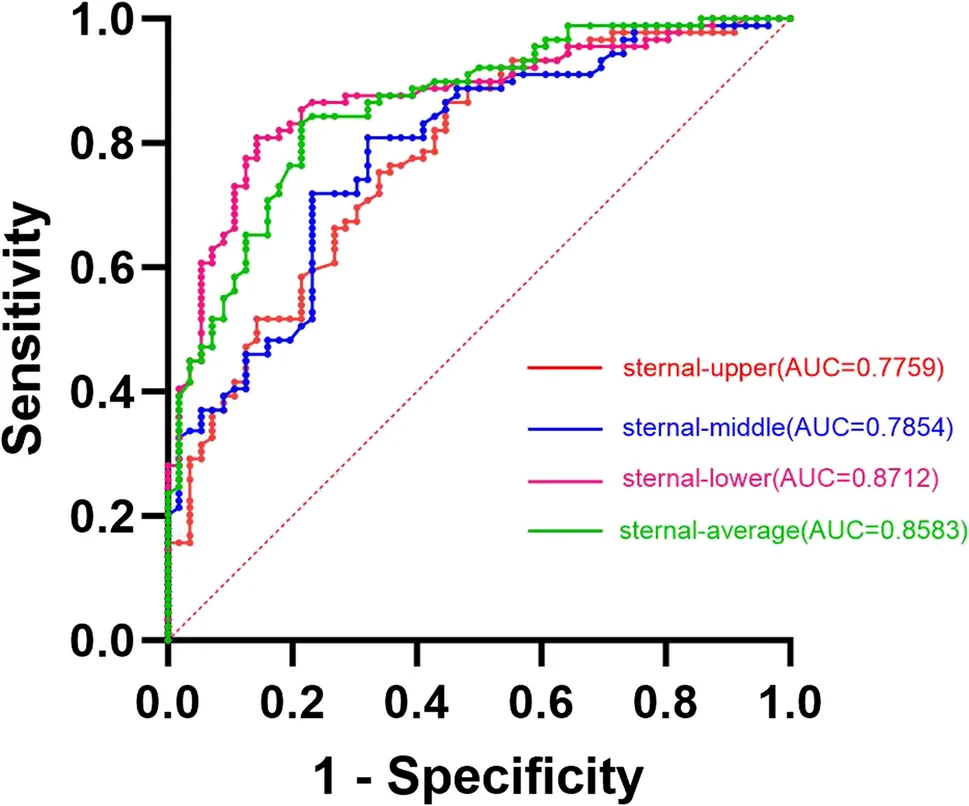
ROC curves demonstrating the diagnostic performance of sternal CT HU values for identifying osteoporosis (osteoporosis vs. non-osteoporosis [normal + osteopenia])

The diagnostic performance of sternal CT HU values at various levels for identifying osteoporosis is summarized in Table 4. All parameters evaluated demonstrated an AUC greater than 0.70, indicating robust diagnostic capability (Fig. 2). Notably, the lower sternal level exhibited the highest diagnostic efficacy, with an AUC of 0.8712 (95% CI: 0.8128–0.9296). Based on the maximum Youden index, the optimal cutoff for the lower sternum was 64.9 HU, yielding a sensitivity of 80.9% and specificity of 85.7% (Youden index = 0.6661). In contrast, the upper sternal level, although having a higher sensitivity (86.5%), was limited by a lower specificity (55.4%).

**Table 4:** Application of ROC curves in evaluating the diagnostic utility of CT values at different levels of the sternum for osteoporosis

Group	AUC (95% CI)	Youden’s index	Balanced threshold	Sensitivity	Specificity
Sternal-upper	0.7759 (0.6986–0.8532)	0.4188	96.20	0.8652	0.5536
Sternal-middle	0.7854 (0.7101–0.8608)	0.4876	75.30	0.8090	0.6786
Sternal-lower	0.8712 (0.8128–0.9296)	0.6661	64.90	0.8090	0.8571
Sternal-average	0.8583 (0.7977–0.9190)	0.6172	77.20	0.8315	0.7857

### Logistic regression analysis

Logistic regression analysis was performed for each sternal level. Consistent with the ROC findings, the lower sternum showed the strongest association with osteoporosis. Table 5 presents the univariate and multivariate logistic regression results for the lower sternal level. In multivariate analysis, after adjusting for age, BMI, and gender, lower sternal HU value was an independent predictor of osteoporosis (adjusted OR = 0.810, 95%CI: 0.750–0.875, *P* < 0.01), while age (OR = 1.045, *P* = 0.162) and gender (OR = 0.810, *P* = 0.675) were not significant in the multivariate model.

**Table 5:** Univariate and multivariate logistic regression analysis for predictors of osteoporosis

Variable	Univariate OR (95% CI)	*P*	Multivariate OR (95% CI)	*P*
Lower-sternal HU	0.802(0.743–0.866)	< 0.01	0.810 (0.750–0.875)	< 0.01
BMI	0.837(0.760–0.923)	< 0.01	0.836 (0.734–0.952)	< 0.01
Age	1.079(1.033–1.127)	< 0.01	1.045 (0.983–1.111)	0.162
Gender	0.658(0.329–1.318)	0.238	0.810 (0.304–2.161)	0.675

## Discussion

This study evaluated the feasibility of using sternal CT HU values as a supplementary method for identifying osteoporosis in elderly individuals undergoing routine chest CT. We observed a significant positive correlation between sternal CT HU values and T-score, consistent with the findings of Sebro et al. [19].These results suggest that sternal CT HU values may serve as a potential marker for opportunistic osteoporosis screening, particularly in settings where DXA is not readily available. Among the evaluated regions, the lower sternal region demonstrated the highest diagnostic performance (AUC = 0.8712), suggesting its potential value for opportunistic osteoporosis screening using routine chest CT.

Sebro et al. [19] used a machine learning–based method implemented in 3D Slicer to perform three-dimensional segmentation of the trabecular bone marrow and quantify sternal CT HU values. Their study demonstrated a significant positive correlation (*p* < 0.05) between sternal CT HU values and DXA-derived T-score, with an AUC of 0.736. These findings provide an important methodological and conceptual support for the present study. Unlike the work by Sebro et al. [19], our study further extended this approach by measuring CT HU values at different anatomical levels of the sternum on routine chest CT scans and directly comparing these measurements with patients’ DXA results. As chest CT is widely applied in cardiopulmonary assessment, cancer follow-up, and other routine clinical practice, it offers a practical opportunity for opportunistic osteoporosis screening [26–28].

Selthofer et al. [29] reported that reduced sternal mineral density is associated with osteoporosis. To date, research on sternal mineralization has primarily focused on cardiac surgery and postoperative complications, partly because DXA cannot reliably assess mineral density in the sternum due to its complex anatomy. Consequently, evidence supporting the use of sternal CT HU values for osteoporosis assessment remains limited. In this context, our study further evaluates the role of sternal CT HU values in osteoporosis assessment.

Pickhardt et al. [23]measured CT HU values at the L1 vertebral body on abdominal CT and identified optimal thresholds for diagnosing osteoporosis using ROC analysis. They reported that an L1 CT HU value of 160 yielded a sensitivity of 90% for detecting osteoporosis, whereas a threshold of 110 HU values achieved a specificity greater than 90%. Using a similar ROC-based approach, our study evaluated the diagnostic performance of sternal CT HU values at different anatomical levels. Among these regions, the lower sternum demonstrated the best diagnostic performance, with an optimal CT HU cutoff value of 64.90 (determined by the maximum Youden index), yielding a sensitivity of 80.9%, a specificity of 85.7%, and an AUC of 0.8712. Differences in cutoff values between the sternum and lumbar vertebrae may reflect anatomical and biomechanical variations between these axial skeletal sites.

Moreover, CT HU value showed significantly better diagnostic performance in the lower sternum than in the upper and middle levels. Selthofer et al. [29] reported no significant difference in mineral density between the manubrium and the mid-body of the sternum. Shevroja et al. [30] reported that trabecular-rich regions are more responsive to changes in BMD. From an anatomical perspective, the lower sternum may contain a higher proportion of trabecular bone within the measurement region, which could make it more sensitive to changes in BMD. It is plausible that this feature partially explains its superior diagnostic performance, although this hypothesis requires further validation.

In addition to anatomical factors, we also examined the relationship between sternal CT HU values and clinical parameters. A significant but weak-to-moderate positive correlation was observed between sternal CT HU values and BMI (*r* = 0.172–0.273, *P* < 0.05). This finding aligns with the established concept that higher body weight exerts a protective mechanical and metabolic effect on bone. Conversely, lower BMI is a well-recognized independent risk factor for osteoporosis, associated with reduced bone mass and increased fracture risk [31, 32]. Nevertheless, the modest strength of this correlation suggests that BMI alone cannot serve as a surrogate for sternal CT attenuation in clinical practice.

In our study, the middle sternal region showed the poorest diagnostic performance, with the weakest correlation with DXA T-score (*r* = 0.506) and a relatively low AUC of 0.7854. This finding may be explained by the anatomical complexity of the middle sternum. As a transitional zone between cortical and trabecular bone and between bone and fibrocartilage, this region exhibits marked structural heterogeneity [33], which may limit the reliability of CT HU value measurement in reflecting systemic bone status. Besides, Duman et al. [22] measured CT HU values in the same sternal region in patients with sternal instability and in those with stable sternums, and reported significantly lower values in the instability group than in the stable group (75.36 ± 13.19 vs. 90.24 ± 12.16 HU). In our cohort, the range of CT HU values observed in patients with low bone mass largely overlapped with the values reported for the instability group in their study. This overlap suggests that reduced sternal CT HU values may reflect compromised bone quality and supports a potential association between sternal CT attenuation and osteoporosis.

Conventional chest CT scans usually extend only to the level of the costophrenic angle and do not include the lumbar spine or the lower thoracic vertebrae. In contrast, the sternum is consistently visualized in its entirety. Previous studies have mainly focused on vertebral CT HU values for BMD assessment [34–39], whereas limited research has evaluated the sternum for this purpose. Owing to its anatomical position, it is consistently included in routine chest CT imaging, making it a practical site for opportunistic osteoporosis screening without additional radiation exposure or dedicated protocols.

Spinal degenerative changes, such as osteophyte formation, facet joint sclerosis, and ligament calcification, are common in middle-aged and older adults [40, 41]. Such degenerative changes may compromise accurate ROI placement, leading to overestimated CT HU values and potentially masking true osteoporosis. In contrast, the sternum is a relatively simple flat bone and is less affected by degenerative changes. Previous studies have suggested that sternal dimensions remain relatively stable after the age of 30 [42]. Collectively, these characteristics may allow sternal CT HU values to better reflect trabecular bone metabolism. This stability may help explain the excellent interobserver agreement observed in our study (ICC > 0.92).

Our study pointed out that there was no statistically significant difference between the osteopenia and osteoporosis group at different sternum levels. This finding suggests that while sternal HU measurement can effectively distinguish normal BMD from abnormal BMD, it has limited capability to differentiate between osteopenia and osteoporosis. This limitation may reflect anatomical characteristics of the sternum as a flat bone, which may be less responsive to moderate changes in BMD compared to the vertebral body. Nevertheless, from the perspective of opportunistic screening—which aims to identify individuals at increased fracture risk before osteoporosis is fully established—both osteopenia and osteoporosis represent categories of reduced bone mass that warrant further clinical evaluation. Therefore, sternal CT HU measurement should be considered a binary screening tool (normal vs. abnormal) rather than a substitute for formal DXA or QCT for disease severity grading. Future studies with larger sample sizes and prospective designs are needed to determine whether refined measurement protocols or combination with other parameters could improve the differentiation between osteopenia and osteoporosis.

It is important to emphasize that sternal CT HU measurement should not be considered a replacement for dual-energy X-ray absorptiometry (DXA). DXA remains the gold standard for the definitive diagnosis of osteoporosis and fracture risk assessment. Rather, sternal HU measurement derived from routine chest CT represents an opportunistic screening marker that can help identify individuals at high risk of osteoporosis. Patients with sternal HU values below the optimal cutoff (e.g., 64.9 HU for the lower sternum) should be referred for confirmatory DXA testing. This approach may improve the detection of undiagnosed osteoporosis in patients undergoing routine chest CT for other clinical indications, without additional radiation exposure or cost.

This study has several limitations. First, this was a single-center retrospective study with a relatively small sample size (*n* = 145) and a small group of patients with normal bone mass (*n* = 10, 6.9%), which may be partly attributable to the inclusion criterion of age ≥ 50 years, as older individuals are more likely to have osteoporosis. The one-month interval between CT and DXA examinations may have introduced temporal bias, and the lack of external validation limits the generalizability of our findings. Second, sternal CT HU values showed limited ability to differentiate between osteopenia and osteoporosis, indicating that this method is more suitable as a binary screening tool (normal vs. abnormal) rather than for disease severity grading. Third, patients with severe spinal degenerative changes were excluded to preserve DXA accuracy, which may limit the applicability of our findings to this clinically relevant subgroup. Fourth, CT measurements were performed by orthopedic surgeons rather than radiologists. Although inter-observer agreement was excellent (ICC: 0.926–0.980), the lack of radiologist input is a limitation. Future studies should include radiologists for validation.

## Conclusion

In conclusion, sternal CT HU values measured on routine chest CT scans are significantly correlated with T-score and show good diagnostic performance for detecting osteoporosis in older adults. Among the evaluated anatomical levels, the lower sternum demonstrated the best diagnostic performance and reliability. Given its consistent inclusion in chest CT scans, lower susceptibility to degenerative changes, and high inter-observer reproducibility, sternal CT HU values—particularly at the lower level—may serve as a potential adjunct for opportunistic osteoporosis screening. However, before clinical implementation, these findings—including the optimal cutoff value of 64.9 HU for the lower sternum—need to be validated in larger, prospective, multicenter studies.

## Data Availability

All data supporting the findings of this study are presented within this article. Additional data will be made available from the corresponding author upon reasonable request.

## Abbreviations

DXA: Dual energy X‐ray
CT: Computed tomography
HU: Hounsfield units
ICC: Intraclass correlation coefficients
BMI: Body mass index
ROC: Receiver operating characteristic
BMD: Bone mineral density
QCT: Quantitative computed tomography
WHO: World Health Organization
ROI: Region of interest
AUC: Areas under the receiver
CI: Confidence interval
